# Impact of the gut microbiota-Th17 cell axis on inflammatory depression

**DOI:** 10.3389/fpsyt.2024.1509191

**Published:** 2024-11-25

**Authors:** Xiuzhi Jia, Jiayi Wang, Dan Ren, Kaibo Zhang, Hongliang Zhang, Tengchuan Jin, Songquan Wu

**Affiliations:** ^1^ Department of Immunology and Pathogen Biology, College of Medicine, Lishui University, Lishui, Zhejiang, China; ^2^ Center of Disease Immunity and Intervention, College of Medicine, Lishui University, Lishui, Zhejiang, China; ^3^ Division of Life Sciences and Medicine, Laboratory of Structural Immunology, University of Science and Technology of China (USTC), Hefei, Anhui, China

**Keywords:** depression, interleukin-17, T-helper 17 cells, gut microbiota, *segmented filamentous bacteria* (SFB)

## Abstract

Depression is a serious cognitive disorder that results in significant and pervasive deficits in social behavior. These deficits can be traced back to the intricate interplay between social, psychological, and biological factors. Inflammatory depression, a treatment-resistant or non-responsive subtype of depression, may be related to the interaction between the gut microbiota and interleukin-17-producing CD4^+^ T cells (Th17 cells). The heterogeneity, plasticity, and effector role of Th17 cells in depression may be influenced by microbiota factors. Commensals-elicited homeostatic Th17 cells preserve the morphological and functional integrity of the intestinal barrier. In addition to pathogen-elicited inflammatory Th17 cells, commensal-elicited homeostatic Th17 cells can become conditionally pathogenic and contribute to the development of inflammatory depression. This review delves into the possible involvement of Th17 cells in inflammatory depression and examines the interplay between gut microbiota and either homeostatic or inflammatory Th17 cells.

## 1 Introduction

Depression is an umbrella term used to describe a range of transient or chronic low-mood disorders. According to recent epidemiological studies, the lifetime incidence of depression in general populations varies from 10% to 33.7% (1). Tremendous preclinical and clinical progress in depression has revealed the complex bidirectional communication between the gut and the brain (2). As the largest endocrine organ in mammals, the gastrointestinal tract can secrete dozens of neural peptides, which can bind cognate receptors on vagus nerve terminals and immune cells to mediate indirect gut-brain communication (3). On the other hand, gut microbiota or microbiota-related metabolites and neurotransmitters can interact with the enteric nervous system (ENS) and vagus nerves, which can function as a fast and direct response route to reach the brain and regulate the neural and inflammatory actions involved in depression (4–6). It is worth noting that some specific microbiota-related metabolites and neurotransmitters (e.g., serotonin, gamma amino butyric acid, tryptophan metabolites, and catecholamines) can penetrate the blood-brain barrier (BBB) and act directly on the receptors in the brain (7).

Treatment for depression often includes a combination of prescription medication and psychotherapy. Selective serotonin reuptake inhibitors, serotonin-norepinephrine reuptake inhibitors, and norepinephrine-dopamine reuptake inhibitors are the most commonly prescribed antidepressants, which only remit in roughly one-third of patients (8). Patients who are not responding to treatment or who are resistant to treatment are considerably more likely to have elevated inflammatory markers, such as tumor necrosis factor (TNF)-α, interleukin (IL)-6, and C-reactive protein (9, 10). Although not all patients with MDD have low-grade inflammation, elevated inflammation is linked to a poor response to the first-line antidepressant treatment (11, 12).

Comorbid depression is a common and debilitating neuropsychiatric symptom exhibited by patients with multiple medical conditions, such as diabetes, inflammatory bowel disease, Alzheimer’s disease, multiple sclerosis, cerebrovascular accidents, and multiple sclerosis (13–15). An etiological trigger of comorbid depression is theorized to be the inflammation within the central nervous system (CNS). The potential connection between gut microbiota-mediated inflammation in the periphery and neuro-inflammatory in the brain is unclear (16). Thus, it is essential to look into the common pathogenic pathways linked to depression and related illnesses to develop more effective treatments.

R.S. Smith first proposed the macrophage-mediated neuro-inflammatory theory of depression in 1991, which provides an explanation for the significant association of depression with stroke, coronary heart disease, rheumatoid arthritis, and other diseases where macrophage activation occurs (17). Michael Maes et al. provide the first evidence of microbiota-gut-brain abnormalities in MDD patients. Elevated blood levels of IgM/IgA against *Morganella morganii* and *Klebsiella pneumoniae* lipopolysaccharides (LPS) have been observed, which may indicate a link between increased gut permeability and active neuroinflammation (18). The gut microbiome typically exhibits changed composition and ectopic colonization in response to both acute and chronic stresses, which can engage with the enteric nervous system and the local immune system to further promote microglial activation, neurogenesis, and behavioral control in the cerebrum (19).

Of the immune cells demonstrated, a certain microbiota can control the generation and development of interleukin-17-producing CD4^+^ T cells (Th17 cells) (**Table 1**). Th17 cells, specific for symbiotic *segmented filamentous bacteria* (SFB), are constitutively home to the lamina propria of the small intestines, which can support healthy gut function and immune surveillance. When compared to Treg cells, Th17 cells prefer to migrate across the BBB through the endothelium to target the CNS neurons. This may be because Th17 cells have different TCR repertoire varieties and chemokine expression (20). Remarkably, Th17 cells in peripheral blood and the gut microbiota in depressed patients are clinically correlated (21).

**Table 1:** Effects of microbiota on T helper cells.

	Treg cells	Th1 cells	Th2 cells	Th17 cells	Key molecules
*Segmented filamentous bacteria*				↑	Serum amyloid A (89)
*Escherichia Coli*	↑			↑	TLR4/NF-κB signaling (99)
*Bifidobacterium adolescentis*				↑	mechanosensitive integrins & cytokines (e.g., TGF-β) (34)
*Staphylococcus aureus*				↑	SEB (100)
*Candida albicans*				↑	ALS3P (101)
*Clostridium cluster IV and XIVa*	↑				TGF-β (102)
*Akkermansia*	↑				short-chain fatty acids (SCFAs) (103)
*Bacteroides*	↑	**↓**		**↓**	Bile acid metabolism (104)
*Lactobacillus*	↑				GM-CSF-macrophage-IL-1β (105, 106)
*Streptococcus*				↑	IL-6 (107)
*Helminth*			↑		Thymic stromal lymphopoietin (108)
*Allobaculum Eggerthella lenta* *Tritrichomonas musculis*		↑		↑ ↑	Cardiac glycoside reductase 2 (31) IL-18 (46)

TLR4, Toll-like receptor 4; NF-κB, Nuclear Factor kappa-light-chain-enhancer of activated B cells; ALS3P, Agglutinin-like sequence 3 protein; TGF-β, Transforming growth factor-β; SEB, Staphylococcus aureus enterotoxin B; SCFAs, Short-chain fatty acids; ALS3P, Agglutinin-like sequence 3 protein; GM-CSF, Granulocyte-macrophage colony-stimulating factor; IL-1β, Interleukin-1β; IL-6, Interleukin-6; IL-18, Interleukin-18. ↑, up-regulated; ↓, down-regulated.

A deeper comprehension of the relationship between Th17 cells and gut microbiota will substantially advance our understanding of inflammatory depression. By discussing the roles played by gut microbiota and Th17 cells in the development of depression, this review will open the door to the diagnosis and treatment of inflammatory depression.

## 2 Th17 differentiation and trans-differentiation related with or without aryl hydrocarbon receptor

Th17 cells have been identified since 2005 and the differentiation of Th17 cells can be defined by the expression of RAR-related orphan receptor-gamma (RORγt), a lineage-specific transcription factor, as well as the production of IL-17A, IL-17F, IL-21, and IL-22. Depending on the cytokine microenvironment, transformation growth factor (TGF)-β1/IL-6-mediated signaling, stimulates the development of homeostatic Th17 cells that have the potential to generate IL-17 but do not easily trigger autoimmune illness without further exposure to IL-23. On the other hand, TGF-β3/IL-6-mediated signaling or IL-6/IL-23/IL-1β-mediated signaling might cause the development of inflammatory Th17 cells (22). The pathogenicity of Th17 mainly relies on the co-production of interferon (IFN)-γ and granulocyte-macrophage colony-stimulating factor (GM-CSF), which is stimulated by IL-23 (23, 24). Chemokine receptor 6 (CCR6), a characteristic chemokine receptor of Th17 cells, can bind to C-C motif chemokine ligand 2 (CCL2) and CCL20 expressed on endothelium, which further facilitates the production of IL-17 and the migration of Th17 cells into the CNS (25).

Aryl hydrocarbon receptor (AHR), a ligand-dependent environmental sensor and transcription factor, can facilitate the recruitment of RORγt to the IL-22 promoter with induced IL-22 expression and promoted Th17 cell development. Although *Arnt* is expressed in all CD4^+^ T cell subsets, AHR is only functional in differentiated Th17 cells. Whatmore, in addition to inhibiting the activation of STAT1 and STAT5, AHR contributes to pathogenic Th17 development by inducing the expression of Aiolos, which silences *il2* expression (26). Additionally, AHR may promote the development of transcriptional modules linked to non-pathogenic Th17 cells and/or the trans-differentiation of Th17 cells into anti-inflammatory Tr1-like cells that produce IL-10 during the resolution of inflammation in tumors (26). All of these findings point to a function for AHR in the early phases of Th17 cell development, when the cells are still producing large amounts of IL-10 and have not yet reached the full extent of their pathogenic potential.

Diverse sources of physiological AHR ligands, such as environmental toxins and microbiota-dependent metabolites and co-metabolites have been discovered. In reality, for Th17 cells to differentiate into completely pathogenic effector cells, constant environmental agent exposure and IL-23 driven microenvironment are required. Well-known high-affinity ligands of AHR include the endogenous ligand of 6-formylindolo[3,2-b] carbazole (FICZ) and the exogenous toxin of 2,3,7,8-tetrachlorodibenzo-p-dioxin (TCDD). FICZ-induced AHR activation exacerbates experimental autoimmune encephalomyelitis (EAE) with increased Th17 cell differentiation and disrupted Treg cell differentiation (27). However, TCDD-induced AHR activation inhibits Th17 polarization and regulates non-eosinophilic airway inflammation in asthma (28). Commensal *Lacobacillus* species produce tryptophan metabolite of indole-3-adehyde (IAld), which maintains intestinal homeostasis and inhibits the growth of pathogenic microorganisms as well as the exacerbation of inflammatory bowel disease (29). All in all, environmental toxins and microbiota-related metabolites may operate on AHR to modify Th17 cell differentiation in a context-specific way.

Through a mechanism independent of AHR, the intestinal microbiota can affect the development and differentiation of Th17 cells. For example, *Actinobacterium Eggerthella lenta* can manufacture the enzyme cardiac glycoside reductase 2 to metabolize RORγt inhibitors, potentially exacerbating inflammation and triggering an enhanced Th17 response (30). Through the p38-MAPK pathway, short-chain fatty acids (SCFAs) like acetate, propionate, and butyrate can promote Th17 cell development and proliferation while hindering their intestinal sequestration. *Eggerthella lenta* can produce 3-oxolithocholic acid (3-oxoLCA) and isolithocholic acid (isoLCA) to inhibit Th17 cell differentiation by inhibiting RORγt (31).

## 3 Tissue-resident homeostatic Th17 cells elicited by commensals and inflammatory Th17 cells elicited by pathogens

Functionally, Th17 cells can be further divided into homeostatic/non-pathogenic cells and inflammatory/pathogenic cells that cause cytokine-skewed immune responses. Mechanically, homeostatic Th17 induction depends on the presence of certain bacteria (SFB, *Bifidobacterium adolescentis*, and *Citrobacter rodentium*) and fungus (*Candida albicans*) within the gut microbiota (32). SFB-mediated Th17 cell differentiation is likely to occur through a mechanism independent of Toll-like receptors, NOD-like receptors, and ATP signaling, but functionally analogous microbes in humans have not been defined (33). On the other hand, *Bifidobacterium adolescentis*, one of the human symbiont bacterial species, alone can induce robust Th17 cell accumulation in the murine intestine (34). Functionally, gut commensal-specific Th17 cells possess an immunoregulatory role and curb effector T cell activity *in vitro* and *in vivo* in an IL-10-dependent and c-MAF-dependent manner (35). In addition to regulating the composition and translocation of the commensal microbiota, homeostatic Th17 cells mediate surveillance and early protection in the mucosa against extracellular bacteria, fungi, protozoa, and viruses. For instance, Th17 cells that are homeostatically produced by SFB do not take part in inflammatory processes (36).

On the other hand, *Citrobacter rodentium*, a naturally occurring mouse-specific pathogen, commonly utilized to model human enteropathogenic *Escherichia coli* infection, can induce IL-23-dependent Th17 cells mediated immune response in the lamina propria. *Citrobacter rodentium*-elicited Th17 cells show extensive plasticity towards pro-inflammatory phenotype and are widely disseminated into the periphery (37, 38). It’s important to note that enterotoxigenic *Escherichia coli*, a major cause of diarrhea in children and travelers in developing countries, induces intestinal IL-17 expression and a prominent Th17 cell response (39). Furthermore, in EAE, autoimmune arthritis, and autoimmune renal disease model, pathogen-specific Th17 cells in the gut can worsen such extra-intestinal inflammation. After being immunized with myelin oligodendrocyte glycoprotein (MOG) peptide, germ-free C57BL6/J mice have dramatically reduced EAE compared to conventionally colonized mice, while the introduction of SFB could promote the development of EAE with enhanced Th17 cell accumulation in the CNS, indicating that gut bacteria can impact neuroinflammation (40). On the other hand, the Th17 cell compartment in the lamina propria is restored when SFB is introduced into germ-free K/BxN mice, which are produced by mating KRN TCR transgenic mice on the B6 background with NOD mice, and this quickly drives the ensued arthritis (41). Depletion of intestinal Th17 cells in antibiotic-treated or germ-free mice could ameliorate the incidence of renal disease, and *Citrobacter rodentium*-elicited Th17 cells can egress from the intestine to the kidney *via* the CCL20/CCR6 axis to induce a more severe renal phenotype (42).

Homeostatic, stem-like TCF1^+^IL-17^+^SLAMF6^+^ Th17 cells are maintained by the microbiota in the intestine. Upon EAE induced by MOG immunization, such homeostatic, stem-like Th17 cells can be converted into encephalitogenic GM-CSF^+^IFN-γ^+^CXCR6^+^ Th17 cells by IL-23 to promote cerebral tissue destruction. All of these indicate that intestinal homeostatic Th17 cells serve as a source from which pathogenic Th17 cells can be transformed to facilitate CNS pathogenesis (24, 43).

## 4 Th17 cell heterogeneity associated with gut microbiota

The concept of Th17 cell heterogeneity originated from analyses demonstrating that different cytokine combinations that generate authentic IL-17-producing Th17 cells in culture have different capacities to induce tissue inflammation upon adoptive transfer (44). The heterogeneity of antigen-specific Th17 cells in humans can be induced by different bacteria, such as SFB, *Escherichia coli*, *Allobaculum Eggerthella lenta*, and *Bifidobacterium adolescentis* (34, 45). On the other hand, rodent parabasalid *Tritrichomonas musculis* activates epithelial inflammasome to induce IL-18 release, which can promote dendritic cell-driven Th17 immunity and confer dramatic protection from mucosal bacterial infections (46).

Of clinical relevance, human Th17 cells demonstrate heterogeneity with different functions and alternative states. Transcriptomic investigation demonstrates that human T cell subtypes are transcriptionally similar to murine *in vitro*-differentiated non-pathogenic and pathogenic Th17 cells. *Staphylococcus aureus*–specific Th17 cells in mice produce IL-17 and IL-10, but no IFNγ, and thus resemble non-pathogenic/homeostatic IL-17^+^IFN-γ^-^IL-10^+^ Th17 cells in humans. In contrast, *Candida albicans*-specific Th17 memory cells in mice resemble pathogenic IL-17^+^IFN-γ^+^IL-10^-^ Th17 cells in humans.

In addition to unbalanced cytokine conditions, a cytokine kinetics switch is also important to induce the heterogeneity of Th17 cells. High levels of IL-17 but not IL-10 are detected in the resting state of *Staphylococcus aureus*–specific Th17 cells, while down-regulated IL-17 and up-regulated IL-10 production are observed in the activated state (44, 47). Gut-resident SFB-specific Th17 cells that express TCF1^+^ can differentiate into intestinal commensal-specific Th17 cells that produce IL-10 and have an anti-inflammatory character shown by the expression of co-inhibitory receptors and IL-10 (35).

## 5 Characteristic gut dysbacteriosis in depression

The person who has consistently elevated levels of lipopolysaccharide (LPS) in the bloodstream, even in the absence of clearly visible peripheral inflammation, may experience depression (48). The Hamilton Depression Rating Scale indicates a strong correlation between the severity of MDD and inconsistent α- and β-diversity of microbiota (49). Based on differences between bacterial taxa, depression is typically linked to a higher abundance of pro-inflammatory species, such as *Enterobacteriaceae* and *Desulfovibrio*, and a lower abundance of bacteria that make SCFAs, such as *Faecalibacterium* (50). Depressed individuals fit into a particular-enterotype microbial landscape with notably low amounts of *Coprococcus* and *Dialister*, according to a major Flemish Gut Flora Project (n=1054). Similar findings have also been confirmed in a different, independent Dutch LifeLines DEEP cohort (n=1070) (51). On the other hand, children and adolescents with depression have changed fungal microbiome taxonomic composition, while fungal diversity remains unchanged (52). Patients with depression also exhibit increased bacteriophage shifts of *Microviridae*, *Siphoviridae*, and *Caudovirales* (53, 54). These all point to a possible connection between depression and altered microbiota.

There is growing evidence linking the diversity of gut microbes to depression in pregnant or nursing mothers and their offspring. *Bifidobacteria* and other beneficial microorganisms are less prevalent in babies whose mothers report higher levels of stress, anxiety, and depression (55, 56). According to prospective cohort research, the α-diversity of pregnant women falls more sharply in those with more severe depressive symptoms. During the later stages of pregnancy, elevated CCL2 levels are associated with maternal depression. The levels of umbilical CCL2 at delivery are inversely correlated with the relative abundance of maternal fecal *Lactobacilli* (57). It is important to remember that early-life stressors, including mother separation, can cause intestinal dysbiosis and alter host physiology, which can subsequently lead to depressive-like behavior. Additional studies show that although gut microbiota is important, it is not enough to cause depressive-like symptoms after mother separation. Both host and microbial factors must work together as significant drivers for neonatal depression to arise (58).

In a single large-scale Netherlands population-based cohort, Mendelian randomization analysis indicates a potential causative link between *Morganella* and MDD, which is in line with the risks identified by 16-year follow-up observation (59). The prevalence and median value of serum IgA/IgM against *Morganella* LPS are much greater in MDD patients than in healthy volunteers, suggesting that serological testing performs significantly better in diagnosing the disease (60). Interestingly, when human-to-mouse and mouse-to-mouse microbiota transfer from a sad donor to a non-depressed recipient, intestinal mucosal permeability and peripheral and cerebral inflammation are raised in recipient mice that display depressive-like behaviors (16, 61, 62). The first evidence that multispecies probiotics could change cognitive reactivity to depressed mood comes from a 4-week *Lactobacillus* and *Bifidobacterium* supplements (63). The notion that additional probiotic bacteria, such as *Enterococcus faecium* and *Pediococcus acidolactici*, may be risk factors for the depression outbreak is also notable. While they do not directly cause depression-like behaviors, *Enterococcus faecium* and *Pediococcus acidolactici* greatly exacerbate anxiety/depression-like behaviors produced by *Escherichia coli* or *Klebsiella oxytoca* due to an increased translocation of LPS into the hippocampus (64, 65).

More generally, substances produced by bacteria, such as neurotransmitters, SCFA, TMAO, indoles, bile acids, choline metabolites, lactate, and vitamins, will have a role in the development of depression. In the clinic, there is a positive correlation between the level of serum TMAO and the severity of depression (66). Additionally, an intestinal metabolite of N-ϵ-acetyllysine can predict medication resistance in depressed patients (67). Deciphering the pathways of gut microbiota will address the clinical value of microbiome-related therapies for depression.

## 6 Th17 cells play a role in the onset of stress

Patients with MDD have higher proportions of Th17 cells in the peripheral blood and higher serum IL-17A production (68–70). Moreover, *in vitro* activation of CD4^+^T cells in generalized anxiety disorder patients shows a deficiency in Th1 and Th2 cytokines and elevated Th17 phenotype (substantially greater secretion of TNF-α and IL-17). Correlation analysis shows that Th17 cells and IL-17A levels are not always correlated with the intensity and duration of depressive symptoms in children, adolescents, and late-life depression. IL-17A levels are associated with cognitive evaluations in late-life depression (71). The clinical symptoms of MDD are linked to elevated levels of IL-17 in the male group and IL-1β, IL-6, and CRP in the female group in young people (72). Other studies also testify that highly detected IL-17A, RORγt, and Th17 cells could be utilized to predict the treatment response in the adult (73–75). All of these imply that age or gender-based stratification should be used in the clinical study.

Elevated Th17 cells are also associated with some chronic illnesses that coexist with depression. Postpartum women, elderly gastric cancer patients, acute ischemic stroke patients, and multiple sclerosis patients with depression have elevated Th17 cells and IL-17A. The link between increased risk of depression and Th17 cell and IL17 levels is also supported by multivariate logistic regression or correlation analysis (76–82). It’s interesting to note that there is a correlation between the number of Th17 cells and neurological impairments in multiple sclerosis patients as well as cognitive impairment, stroke recurrence, and mortality. All of the results point to the possibility of treating people with comorbid depression by focusing future research on targets based on Th17 cells.

Corroborating data from mouse research attests to the deleterious effects of Th17/IL-17A in depression. In mice or rats subjected to LPS, acquired helplessness, or unpredictable chronic restraint stress (UCRS), there are more Th17 cells detected in the brains (83–85). Following adoptive Th17 or Th1 cell transfer, only Th17 cell transfer can produce learned-helplessness, even if both Th1 and Th17 cells are increased in the hippocampal regions of learned-helplessness animals. Additionally, the susceptibility to learned helplessness may be increased by adoptively transplanting Th17 cells into Rag2^-/-^ mice lacking endogenous T cells. Furthermore, using the RORγt-GFP reporting system, it is discovered that endogenous central Th17 cell induction is necessary for donor Th17 cells to have pro-depressive properties. All of these suggest that whereas central and peripheral Th17 cells are required to trigger stress-induced depressive-like behavior, Th1 and Treg cells are insufficient to either promote or prevent the development of learned helplessness (86).

Hippocampal Th17 cells in the learned-helpless mice show a similar phenotype of Tfh-17-like cells, characterized by increased expression of follicular cell markers (CXCR5, PD-1) and pathogenic Th17 cell markers (CCR6, IL-23R). It is further revealed that CCR6 knockout or deletion can block transferred Th17 cells from promoting learned helplessness. Functionally, CCR6 deletion is linked to higher programmed cell death protein 1 (PD-1) expression in CCR6-deficient Th17 cells. While CCR6 deletion does not affect the accumulation of Th17 cells in the hippocampus (86). Considering that PD-1 is known to generate anergy in Th17 cells, these results point to a possible mechanism by which CCR6 regulates the pathogenicity of Th17 cells in learned helplessness animals.

In UCRS mice, deletion of CD4-specific AHR or RORγt does not influence behaviors that resemble anxiety or depression. Stressed CD4-specific AHR mutant mice have more RORγt^+^ cells in their lamina propria (87). These all point to the lack of a prominent role for either RORγt or CD4-specific AHR in the development of stress-induced depressive-like behaviors. Conversely, rats who get a prophylactic injection of SR1001 do not exhibit depressive-like behaviors brought on by UCRS. SR1001 can competitively bind to RORα and RORγt, preventing Th17 cells from differentiating and functioning. Interestingly, IL-17 and CCL2 release both markedly increase in the first week of UCRS exposure and then progressively fall over time, while Th17 cell numbers in the dorsal striatum steadily increase and peak in the fourth week (84). However, IL23-stimulated GM-CSF-producing Th17 cells in the CNS can change their surface signature from CCR6 to CCR2 with a still unknown mechanism (88). AHR or RORγt genetic impairment in CD4^+^T cells does not eliminate depression vulnerability in UCRS mice, whereas neutralization of RORα and RORγt may prevent depression in UCRS rats. The distinct functions of Th17 cells in UCRS mice and rats highlight the complexity of the pathophysiology of depression, an issue that needs more research in the future.

## 7 Th17 and gut microbiome interact to exacerbate depression

**Figure 1:**
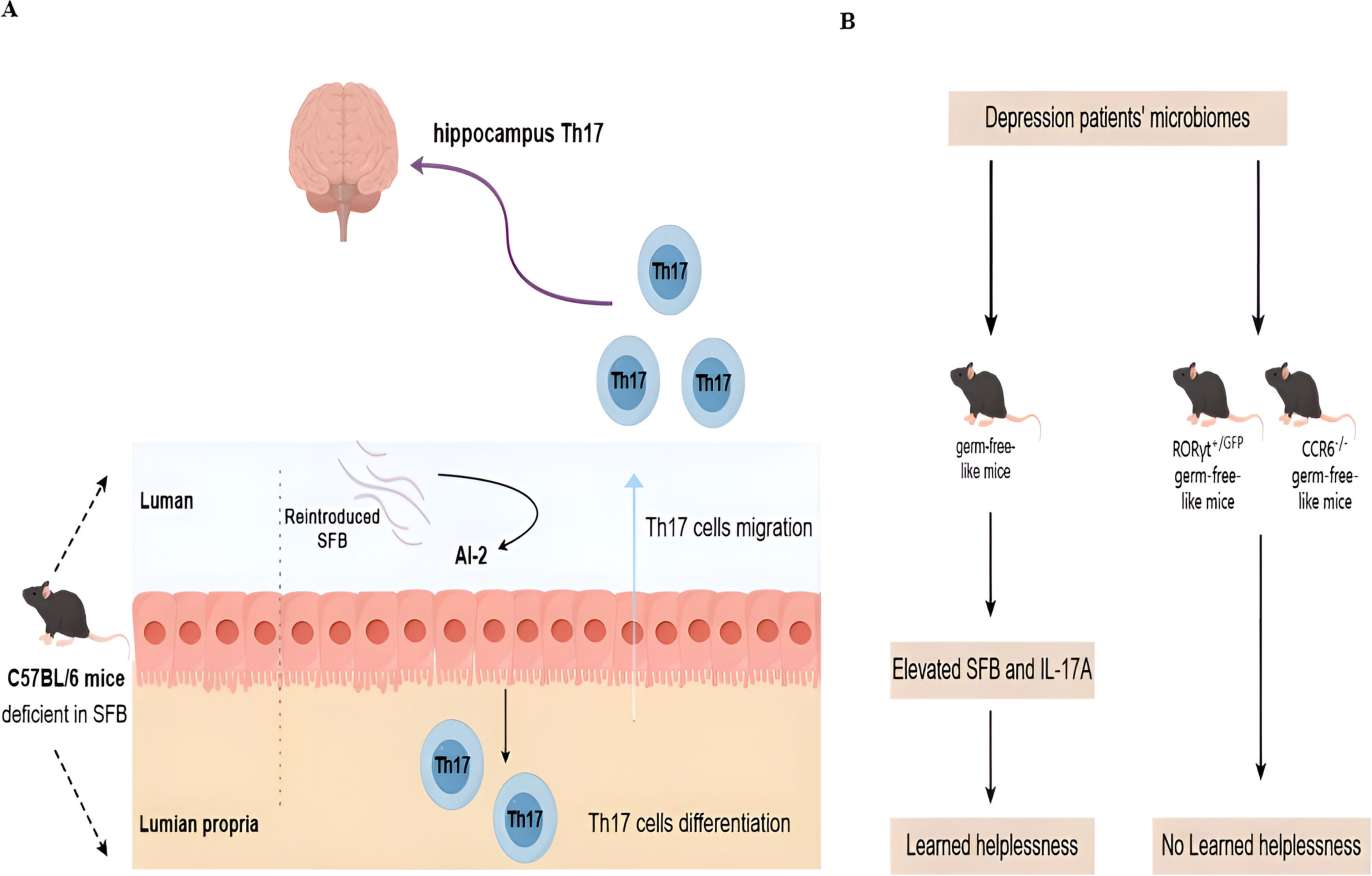
Novel molecular mechanisms for microbiota and Th17 interaction in depression. **(A)** In healthy mice, Th17 cells are only present in the lamina propria of the small intestines in the presence of commensal microbiota in the lumen. Without *Segmented filamentous bacteria* (SFB), Th17 cells are absent. Deficient SFB is observed in C57BL/6 mice derived from Jackson Laboratory. Such mice are resilient to the induction of depressive-like behavior after the re-introduction of SFB in the gut. Mechanically, stress can induce SFB-mediated quorum sensing AI-2 production to promote the differentiation and migration of Th17 cells in the hippocampus and the occurrence of depressive-like behavior. **(B)** The microbiome is sufficient to confer depressive-like behaviors, as demonstrated by the fact that fecal transplants of the microbiomes of human depression patients into germ-free-like mice are sufficient to reduce sociability and increase sensitivity to the learned helplessness paradigm. This microbial effect is dependent on the presence of Th17 cells in the recipient, as germ-free-like recipient mice deficient in Th17 cells (RORγT^+/GFP^ and CCR6^−/−^ mice) are resistant to the behavioral changes induced by the microbiome derived from depression patients.

In addition to clinical connections, there is evidence that Th17 cells and gut microbiota are linked to depression. Mice with active depressive episodes have higher levels of SFB and IL-17 in their feces (89). Microbiota can stimulate peripheral immune cells and promote the migration into the CNS. For example, C57BL/6 mice derived from Jackson Laboratory (deficient in SFB) are resilient to the induction of depressive-like behavior in the learned helplessness paradigm with the re-introducing of SFB in the gut. Mechanically, SFB colonization results in the production of quorum sensing molecule autoinducer-2 and serum amyloid A (SAA)1/2, which in turn act on gut dendritic cells to promote the differentiation and migration of Th17 cells in the hippocampus and the occurrence of depressive-like behavior (**Figure 1A**) (89). In another fecal transfer experiment, germ-free-like mice that are transferred with the microbiomes of depression patients show increased prevalence of SFB and expression of IL-17A. A Th17-dependent imparting impact on sociability impairment and acquired helplessness is seen in RORγt or CCR6-depleted germ-free-like recipient mice (**Figure 1B**) (90).

Pregnant mice inoculated with human commensal bacteria or mouse commensal SFB are more likely to have offspring with neurodevelopmental defects (91). Depression susceptibility can be markedly increased by species from *Morganella*, *Mycobacterium neoaurum*, *Bacteroides* (e.g., *B. thetaiotaomicron*, *B. fragilis*, and *B. uniformis*) (92). Furthermore, a decrease in species that create SCFAs—a crucial metabolic regulator for the generation of inflammatory Th17 cells—is negatively correlated with the severity of depression (93, 94). At the same time, shifts in the composition of gut microbiota in MDD lead to changes in the microbial metabolome, which plays a role in the pathogenesis of MDD. Moreover, microbiota metabolites, such as 4-hydroxyphenylpropionic, 4-hydroxyphenylacetic acid, and caffeic acid, can activate AHR to modulate the Th17/Treg imbalance, strengthening resistance to stress-induced anxiety- and depressive-like behaviors (27, 95). However, within the complex structure of the gut microbial community, it is likely that various microbial species collaborate to produce depression. Understanding the relationship between Th17 cells and the gut microbiota can help us better understand depression.

## 8 Prospects

It is challenging to understand the pathophysiology of depression because different depression symptomatologies cannot be explained by a single hypothesis. Without addressing the causal nature, the data derived from depression patients are mostly descriptive and only present a few likely candidates. The interaction between gut microbiota and inflammatory Th17 cells can cause primary inflammatory depression and comorbid depression. It is still unknown if pro-inflammatory Th17 cells are trans-differentiated tissue-resident homeostatic Th17 cells or whether pro-inflammatory Th17 cells are produced *de novo* from naïve CD4^+^T under pro-inflammatory settings.

It is noteworthy that inflammation regulation may be crucial in the convergence of Th-17 cell-mediated autoimmune response and concomitant depression. In psoriasis patients with co-occurring depression, neutralizing antibodies that target Th17 cells (anti-IL-17A, Ixekizumab; anti-IL-12/IL-23, Ustekinumab) have shown promising benefits (96, 97). Furthermore, the U.S. Food and Drug Administration (FDA) has approved ketamine as an adjuvant treatment for treatment-resistant depression in adults, and ketamine metabolites can functionally inhibit the development and proliferation of Th17 cells (98). Limited information is available on how these findings may translate to depression involving the gut and brain axis. Therefore, there is a great deal of therapeutic potential in focusing on pathogenic Th17 cell activities.
